# Apatinib combined with temozolomide treatment for pseudoprogression in glioblastoma: A case report

**DOI:** 10.1097/MD.0000000000032156

**Published:** 2022-12-09

**Authors:** Mingming Zhao, Haodong Ma, Peng Cheng, Hongjie Yang, Yang Zhao, Qian Han

**Affiliations:** a First Ward of Cancer Center, People’s Hospital of Henan University, Zhengzhou, China; b First Ward of Cancer Center, Henan Provincial People’s Hospital, Zhengzhou, China.

**Keywords:** apatinib, case report, glioblastoma, imaging, pseudoprogression, temozolomide

## Abstract

Glioblastoma is the most common malignant tumor of the central nervous system, which originates from glial cells and corresponding precursors. Due to its strong invasion and rapid growth, the prognosis of patients after treatment is very poor and easy to relapse.

**Patient concerns:**

In August 2015, a 48 years old man with a relapse of glioblastoma.

**Diagnoses:**

The patient was diagnosed by computed tomography, magnetic resonance imaging, and pathological biopsy in this case report.

**Interventions:**

The patient underwent 2 surgeries, radiotherapy, and multiple regular chemotherapy sessions over the next 6 years. Apatinib, an inhibitor of vascular endothelial growth factor receptor 2 was given to treat recurrent glioma.

**Outcomes:**

It was found that radiotherapy combined with temozolomide administration often increased the size of the original lesion or produced a new glioblastoma lesion. The lesion development was similar to tumor progression, which was called pseudoprogression. And it significantly prolonged the survival of this patient.

**Lessons:**

Surgery, radiotherapy and chemotherapy with apatinib and temozolomide are effective to treat the patients with pseudoprogression in glioblastoma.

## 1. Introduction

Glioblastoma is the most common primary malignant tumor of the central nervous system, which is originated from glial cells and corresponding precursors.^[1]^ It is divided into grade I, II, III and IV according to the grading standard of the World Health Organization. Among them, grades I and II are called low-grade glioblastoma, and grades III and IV are called high-grade glioblastoma.^[2]^ The treatment of glioblastoma is mainly surgical resection, and radiotherapy and chemotherapy are the most important comprehensive treatments.^[3]^ The median survival of glioblastoma is over 14 months in patients who are treated following the standardized STUPP regimen (surgery plus temozolomide-based adjuvant chemotherapy, as well as radiotherapy).

Surgical resection can alleviate intracranial hypertension and compression symptoms, reduce tumor load, improve quality of life, and prolong survival. After radiotherapy and chemotherapy, enhanced magnetic resonance imaging (MRI) is routinely used to evaluate the efficacy. The follow-up imaging changes mainly include non-progression, early progressive disease, recurrence, radionecrosis, and so on. Early progression includes pseudoprogression (PSP) and true progressive disease.^[4]^ Pseudoprogression is a treatment-related reaction, which can shrink or disappear without treatment, and the prognosis is good.^[5]^ Here, we reported a rare case with PSP in glioblastoma.

## 2. Case presentation

On August 2 ,2015, a middle-aged (48 years) man was presented with no cause of dizziness, accompanied by hearing loss in the right ear that was more pronounced after exertion. On August 26 in 2015, he underwent surgery for right temporal lobe occupancy, and the postoperative pathology showed glioblastoma. Gamma Knife treatment was performed in another hospital, followed by 3 cycles of chemotherapy with oral temozolomide in our hospital. Then, we evaluated his body condition via imaging examination, which suggested the possibility of recurrence. Therefore, the patient underwent another surgical resection on January 19, 2016 in our neurosurgery department, and the standard treatment for glioma (i.e., 6 cycles of temozolomide treatment with synchronous radiotherapy and chemotherapy) was performed without any adverse effects.

**Figure 1.**
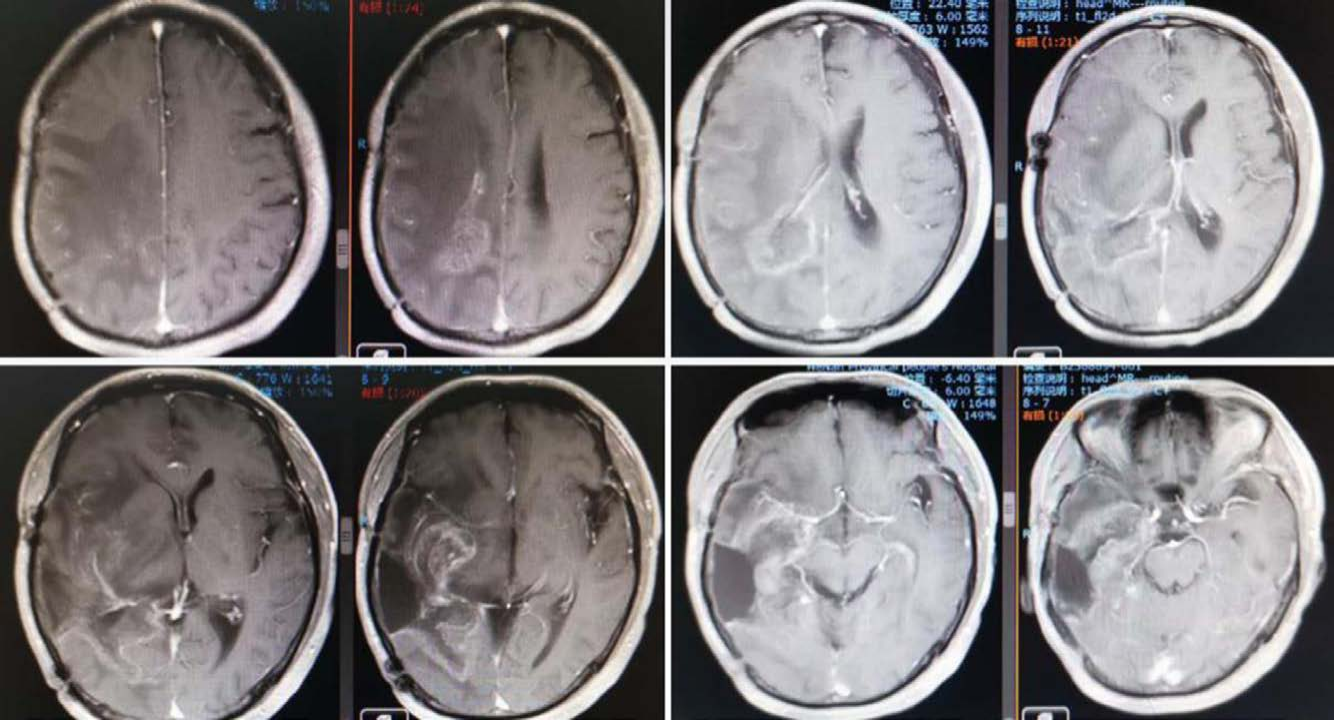
On December 14, 2016, the magnetic resonance imaging of the head showed postoperative changes of glioma, abnormal enhancement of the right temporo-occipital lobe, and possible recurrence.

**Figure 2.**
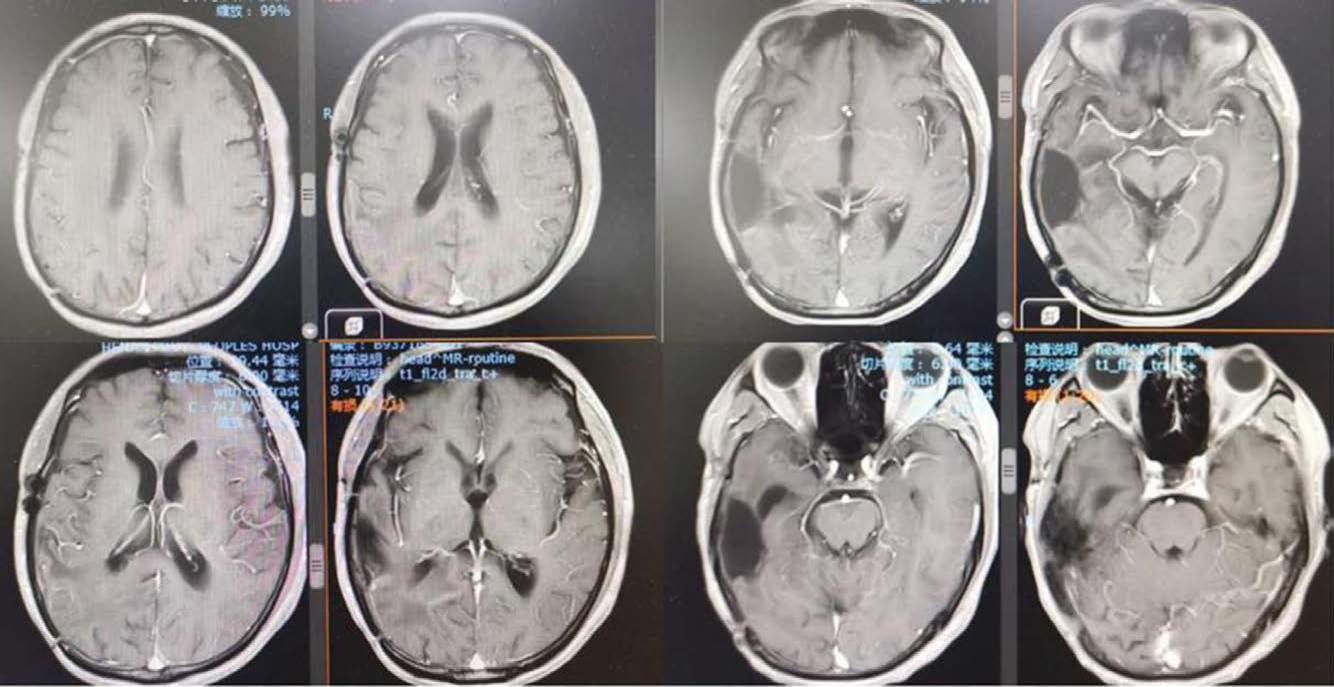
On January 10, 2017, a reexamination of the head magnetic resonance imaging showed that the condition was stable.

**Figure 3.**
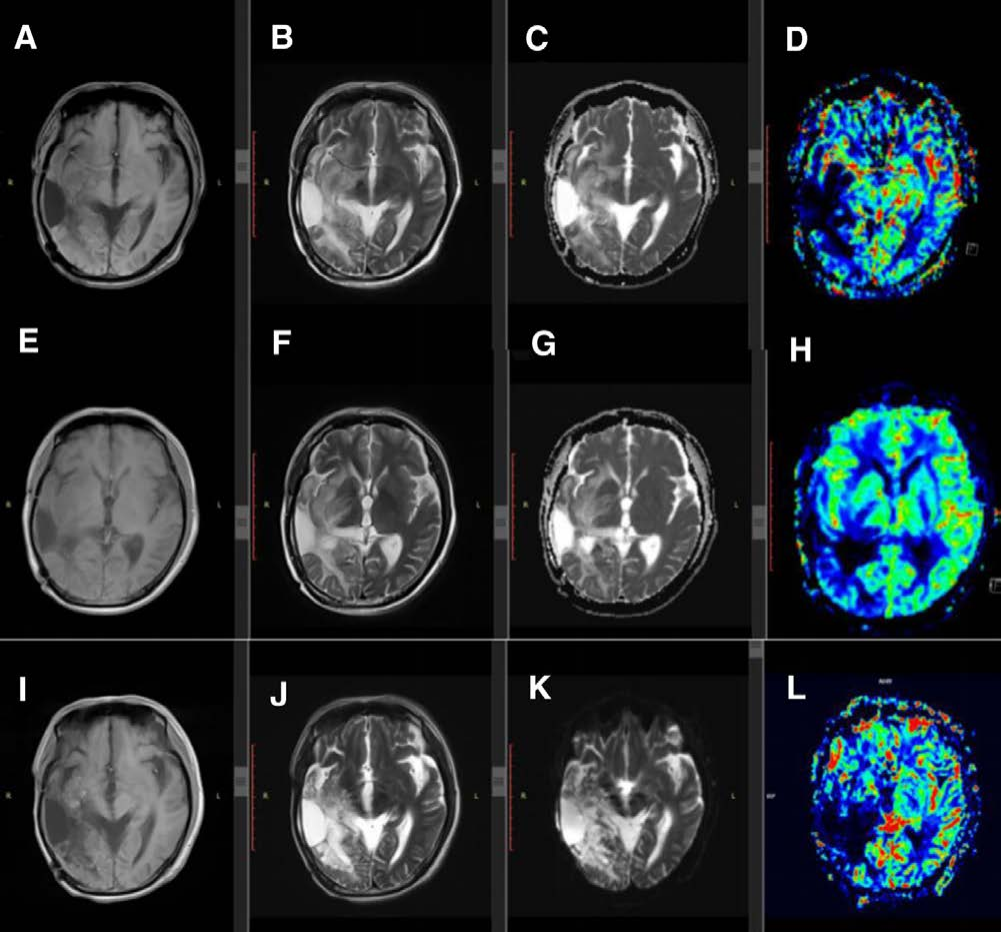
Magnetic resonance imaging on July 10, 2017. (A: T1W; B: T2W; C: ADC; D: PWI). On January 02, 2018 MRI (E: T1W; F: T2W; G: ADC; H: PWI). On May 12, 2020, MRI (I: T1W; J: T2W; K: ADC; L: PWI). PWI = perfusion weighted imaging.

On December 14, 2016, a cranial MRI showed that the postoperative changes were associated with the glioma, such as abnormal enhancement of the right temporo-occipital lobe compression, and possible recurrence (Fig. 1). Pathological biopsy is the gold standard for the diagnosis of postoperative relapsed glioma or pseudoprogression, but its invasive nature limits its clinical application.^[6]^ According to the clinical guidelines, the administration of bevacizumab alone or in combination with a chemotherapeutic agent (lomustine, carmustine, temozolomide, carboplatin, etc.) is recommended to prevent recurrence or PSP.^[7]^ However, bevacizumab is expensive for the patient, and previous administration of lomustine shows poor outcomes. Hence, 6 cycles of apatinib combined with temozolomide treatment with a preferential policy was provided, and regular follow-up examinations were performed via imaging. This disease was found to be stable after 3 cycles of chemotherapy (Fig. 2). On July 10, 2017, it was found that there were several enhancement signal changes in imaging. Multimodal MRI suggested that the disease was stable (Fig. 3). Therefore, the medication was carefully adjusted to prevent the side effects of hypertension and hand-foot syndrome during the treatment, so as to improve the quality of life of the patient and ensure the longer survival.

## 3. Discussion

Glioma is the most common primary intracranial tumor, and its 5-year mortality is second only to pancreatic cancer and lung cancer.^[1]^ The main factor leading to the high mortality of glioma is the high recurrence rate of tumor. The cause of gliomas is unknown and cannot be prevented. Gene abnormalities are detected in some chromosomes, which might influence cancer progression.^[2,3]^ However, the cause of those abnormalities is undetermined. Many researchers have analyzed the genetic, familial, occupational, and environmental factors for identifying the associations among these factors and gliomas in various cases.^[8]^ Environmental risks have been extensively investigated, but no direct factors have been examined.^[9]^ Many cerebral cancers do not exhibit hereditary characteristics, and only 5% of them might be due to genetic background.

The PSP of gliomas is the damage to the brain that occurs after radiotherapy, instead of the actual progression of the glioma. The pathological changes comprise the increased expression levels of tumor necrosis factor-*α* in brain tissue and vascular endothelial growth factor, which increase the permeability of small blood vessels, leading to brain edema, chronic inflammatory responses and structural damage to neurons.^[10]^ PSP is closely associated with the promoter methylation status of isocitrate dehydrogenase and O6-methylguanine-DNA methyltransferase (MGMT). Generally, patients with isocitrate dehydrogenase 1 mutation and MGMT promoter methylation are more prone to PSP and have a better prognosis.^[11]^ Pathological diagnosis is the gold standard for PSP of glioma. Additionally, MRI functional imaging, PET (11C-methionine, 18F-tyrosine), and other imaging methods perform auxiliary diagnosis to identify tumor progression or recurrence.^[4,12,13]^ Thus, it is important to identify PSP and true recurrence for treatment.

The relative cereal blood flow and volume (rCBF and rCBV) in the perfusion weighted imaging sequence reflect the degrees of microvascular neovascularization and perfusion status from different angles.^[14]^ rCBV and rCBF are higher in the case of recurrence. As the main pathological manifestation of pseudoprogression is edema without neovascularization, hypoperfusion is associated with low rCBV and rCBF in perfusion weighted imaging.^[14]^ If MRI cannot differentiate between PSP and true recurrence, multimodal MRI was performed to exclude the possibility of recurrence to avoid misdiagnosis and mistreatment.

For this patient, the anti-angiogenic drug, apatinib, significantly increased the patient’s lifespan. Besides mesenchymal degeneration, cell proliferation, and necrosis, glioblastomas show a significantly increased vascular permeability and microvascular growth.^[2]^ Apatinib is highly efficient in inhibiting angiogenesis, and the vascular endothelial growth factor signal is a representative critical treatment target. Specifically, vascular endothelial growth factor receptor 2 plays an essential role in regulating angiogenesis.^[15]^ Atinib is an oral small molecule tyrosine kinase inhibitor, which can selectively bind and inhibit vascular endothelial growth factor receptor 2, so as to improve the efficacy of traditional antitumor drugs (verapamil, vincristine, adriamycin) and eliminate multidrug resistance.^[16,17]^ In a preclinical study, apatinib has the effect to suppress glioma cell proliferation and migration, and also to promote the anticancer effect of temozolomide.^[18]^ Atinib treatment may be slightly toxic, resulting in anorexia (5.8%), hypertension (5.8%), thrombocytopenia (4.3%) and leucopenia (3.6%) in patients with grade III/ IV tumors.^[18]^ Many of these toxic effects occur during early treatment and can be relieved by reducing the dose of apatinib and performing symptomatic management. Our results suggested that treatment with apatinib and temozolomide is effective and might be better than single temozolomide treatment, specifically for cases with MGMT non-methylation. Thus, apatinib has some advantages for treating relapsed glioma cases.

The long-term imaging follow-up of the patient indicated long-term stability of the MRI performance without tumor progression. Imaging also confirmed the recovery of progression. This suggested that the drug was effective, and despite the occurrence of the side effects of the drugs, such as hypertension, the patient had a good quality of life after our aggressive symptomatic management. Other treatments for recurrent of high-grade glioma included tumor treating fields, immunotherapy, and so on. A series of clinical trials demonstrated that tumor treating fields is as efficient and effective as salvage chemotherapy, and has significantly fewer adverse effects than salvage chemotherapy.^[19]^ Immunotherapy (e.g., PD-1/PD-L1) are also being conducted. Therefore, many other therapeutic methods can be applied to increase the lifespan of patients with recurrent glioma and reduce the side effects of treatment.

## 4. Conclusion

Glioblastoma is highly aggressive and prone to recurrence. In a case report, the patient with glioma was treated with 6 cycles of temozolomide simultaneously with radiotherapy. Within 3 months of treatment, he developed PSP, which was similar to recurrence. Multimodal MRI was used in differential diagnosis. The small-molecule anti-angiogenic drug, apatinib was used to treat the patient with recurrent or PSP glioma and receive the same benefits as treatment with bevacizumab. Additionally, electric field therapy and immunotherapy are other therapeutic strategies that need to be investigated for treating patients with relapsed glioma.

## Author contributions

**Conceptualization:** Mingming Zhao.

**Data curation:** Mingming Zhao.

**Formal analysis:** Mingming Zhao, Peng Cheng.

**Resources:** Haodong Ma.

**Writing – original draft:** Mingming Zhao, Peng Cheng.

**Writing – review & editing:** Hongjie Yang, Yang Zhao, Qian Han.

## References

[1] Zuniga RM Torcuator R Jain R. Efficacy, safety and patterns of response and recurrence in patients with recurrent high-grade gliomas treated with bevacizumab plus irinotecan. J Neurooncol. 2009;91:329–36. 18953493 10.1007/s11060-008-9718-y

[2] Louis DN Perry A Reifenberger G. The 2016 World Health Organization classification of tumors of the central nervous system: a summary. Acta Neuropathol. 2016;131:803–20. 27157931 10.1007/s00401-016-1545-1

[3] Cantidio FS Gil GOB Queiroz IN. Glioblastoma-treatment and obstacles. Rep Pract Oncol Radiother 2022;27:744–53. 36196416 10.5603/RPOR.a2022.0076 PMC9521695

[4] Sun YZ Yan LF Han Y. Differentiation of pseudoprogression from true progressionin glioblastoma patients after standard treatment: a machine learning strategy combinedwith radiomics features from T1-weighted contrast-enhanced imaging. BMC Med Imaging. 2021;21:17. 33535988 10.1186/s12880-020-00545-5 PMC7860032

[5] Sidibe I Tensaouti F Roques M. Pseudoprogression in glioblastoma: role of metabolic and functional MRI-systematic review. Biomedicines 2022;10:285. 35203493 10.3390/biomedicines10020285 PMC8869397

[6] Cao Y Zhang L Wang Y. Antitumor activity of Cedrelone in temozolomide-resistant human glioma cells is accompanied by mitochondrial mediated apoptosis, inhibition of angiogenesis, cell cycle disruption and modulation of ERK/MAPK signalling pathway. J BUON. 2019;24:1204–9. 31424680

[7] Qi A Han J Jia F. 3175 and miR-134 affect proliferation, invasion and apoptosis of glioma cells through PI3K/AKT signaling pathway. J BUON. 2019;24:2465–74. 31983121

[8] Wu XF Liang X Wang XC. Differentiat*ing hi*gh-grade glioma recurrence from pseudoprogression: comparing diffusion kurtosis imaging and diffusion tensor imaging. Eur J Radiol. 2021;135:109445. 33341429 10.1016/j.ejrad.2020.109445

[9] Taal W Oosterkamp HM Walenkamp AM. Single-agent bevacizumab or lomustine versus a combination of bevacizumab plus lomustine in patients with recurrent glioblastoma (BELOB trial): a randomised controlled phase 2 trial. Lancet Oncol. 2014;15:943–53. 25035291 10.1016/S1470-2045(14)70314-6

[10] Hanif F Muzaffar K Perveen K. Glioblastoma multiforme: a review of its epidemiology and pathogenesis through clinical presentation and treatment. Asian Pac J Cancer Prev. 2017;18:3–9. 28239999 10.22034/APJCP.2017.18.1.3 PMC5563115

[11] Strauss SB Meng A Ebani EJ. Imaging glioblastoma posttreatment: progression, pseudoprogression, pseudoresponse, radiation necrosis. Radiol Clin North Am. 2019;57:1199–216. 31582045 10.1016/j.rcl.2019.07.003

[12] Zikou A Sioka C Alexiou GA. Irradiation necrosis, pseudoprogression, pseudoresponse, and tumor recurrence: imaging challenges for the evaluation of treated glioma*s*. Contrast Media Mol Imaging. 2018;2018:6828396. 30627060 10.1155/2018/6828396 PMC6305027

[13] Li H Li J Cheng G. IDH mutation and MGMT *p*romoter methylation are associated with the pseudoprogression and improved prognosis of glioblastoma multiforme *patients who have un*dergone concurrent and adjuvant temozolomide-based chemoradiotherapy. Clin Neurol Neurosurg. 2016;151:31–6. 27764705 10.1016/j.clineuro.2016.10.004

[14] Kim JY Park JE Jo Y. Incorporating diffusion- and perfusion-weighted MRI into a radiomics model improves diagnostic performance for pseudoprogression in glioblastoma patients. Neuro Oncol 2019;21:404–14. 30107606 10.1093/neuonc/noy133 PMC6380413

[15] Ferrara N. The role of VEGF in *the regulati*on of physiological and pathological angiogenesis. Exs 2005:209–31. 15617481 10.1007/3-7643-7311-3_15

[16] Tong XZ Wang F Liang S. Apatinib (YN968D1) enhances the efficacy of conventional chemotherapeutical drugs in side population cells and ABCB1-overexpressing leukemia cells. Biochem Pharmacol. 2012;83:586–97. 22212563 10.1016/j.bcp.2011.12.007

[17] Mi YJ Liang YJ Huang HB. Apatinib (YN968D1) reverses multidrug resistance by inhibiting the efflux function of multiple ATP-binding cassette transporters. Cancer Res. 2010;70:7981–91. 20876799 10.1158/0008-5472.CAN-10-0111 PMC2969180

[18] Ge J Li C Xue F. Apatinib plus temozolomide: an effective salvage treatment for recurrent glioblastoma. Front Oncol. 2021;10:601175. 33634023 10.3389/fonc.2020.601175 PMC7901881

[19] Stupp R Wong ET Kanner AA. NovoTTF-100A versus physician’s choice chemotherapy in recurrent glioblastoma: a randomised phase III trial of a novel randomised phase III trial of a novel treatment modality. Eur J Cancer. 2012;48:2192–202. 22608262 10.1016/j.ejca.2012.04.011

